# Book Notices

**Published:** 1855-09

**Authors:** 


### BOOK NOTICES.

*Proceedings of the Connecticut Medical Society for* 1855.

THIS pamphlet shows a strong, quiet, comfortable sort of a profession in the "land of steady habits." There are some few contributions of scientific interest, but a large portion of the document is filled up with biography, lists of members, and other matters that concern not scientific progress; but the details of internal organization. The State Society of Connecticut does as well as those of other states, but we find one grave fault with the proceedings of nearly all these bodies.—There is nothing carried through in an energetic, comprehensive and effective manner, but the whole budget of "proceedings" is a medley consisting in the better part, of monographs by individual members, and in the less valuable portions, of local observations rendered worthless by the absence of any general system of co-operation, of puffs on dead members, of dry inaugural discourses, and of voluminous records of names, residences, motions, votes, by-laws, constitutions, etc. Much of this kind of matter, must of course come in, but the scientific part should be better elaborated, and the rest more condensed. Active medical men, have had a herculean labor in getting these Societies into a substantial existence: there now remains one thing more, and that is to get them properly at work. It is true, the bare existence of a state society is useful to the physicians who compose it, by promoting good feeling; but the organization should have a higher function. *It should do something.* Every state society ought to have not only a department of monographs, but also a scheme of connected observations, which shall settle the topography of diseases, and preserve to the world a yearly record of their medical statistics. Such materials collected in every State, would furnish matter for reference forever.

We notice that several societies have undertaken to establish meteorological observations. We suggest that this work, though important, is too difficult for any society to carry out over its whole territory, consequently it had better be confided to the care of the Smithsonian Institute, who will do it far more thoroughly, and from whose records, copies of observations can be obtained, either for the archives of the societies. or for the use of members who wish to develop the relations of climate and disease.

"Search out the secrets of Nature." The Annual Discourse before the Massachusetts Medical Society, at Springfield, June 27, 1855. By Augustus A. Gould, M. D.

This oration, from its style, appears to have been delivered before a popular audience, consequently it makes no pretensions to profundity. As a popular address it is well adapted to establish in the minds of an audience, sound and common sense views of their relations to their medical advisers We gather from the address that the society gives zest to its meetings by a good, hearty, jovial supper, an excellent plan provided they avoid the extravagant, and otherwise objectionable revels, which formerly closed the sessions of the American Medical Association.

A Practical Treatise on the Diseases, Injuries and Malformations of the Urinary Bladder, the prostate Gland and the Urethra. By S. D. Gross, M. D., Prof. of Surgery, in the University of Louisville, &c., &c. ; Second Edition, revised and much enlarged, with one hundred and eighty-four illustrations. Philadelphia : Blanchard & Lea, 1855.

Dr. Gross in the new edition of this work, has added upwards of two hundred pages of original matter. The author regrets that the size of the book is thereby increased for says he, "a large book is undoubtedly a great evil."

We cannot pretend to give a review of this work, although there is much of interest in its pages. The treatment as detailed in some of the cases we think open to criticism. Leeches, purgation, cauterization, &c., are used unsparingly, and as we think in some cases where tonics instead of antiphlogistics are required.

The author thinks that puncture of the bladder is rarely necessary,—he having never performed it. In reference to this operation he says:

From what I have seen of retention of urine, I am satisfied that puncture of the bladder is rarely, if ever necessary. It is only in cases of extensive enlargement of the prostate gland, attended with great tenderness and swelling of the surrounding parts; in laceration of the urethra and infilteration of urine into the scrotum; and in deep-seated, impassible stricture, that the operation should ever be seriously thought of. All other forms of retention will, there is reason to believe, yield to the catheter, aided by time and by soothing measures. The celebrated Desault, indeed, used to maintain that puncture of the bladder was never necessary, on the ground that there was no case in which a skilful practitioner could not reach this organ with a catheter. During the eight years in which he held the rank of chief surgeon to the Hotel-Dieu, in Paris, he performed the operation only once, and that was soon after he took charge of the institution. It is true some of his cotemporaries have declared that he trusted too implicitly in his dexterity, and that he occasionally made a false passage; but this may have been a mere imputation, raised, without any just foundation, by his detractors. Be this as it may, no one, who has any experience on the subject, can doubt that the operation has often been performed unnecessarily, and that those who have most frequently executed it have been young men, little versed in the use of the catheter and bougie. The late Mr. Liston, whose experience in the treatment of urinary affections must have been very extensive, states that he never punctured the bladder but once.

We have recently had occasion to puncture the bladder above the pubes. The details of the case, we may report at some future time. The operation has been followed by no unfavorable symptoms. From the statistics of Mons. Mondiere, it appears that of nine cases of puncture in the perineum the operation was successful in six; of twenty-eight cases of recto-vesical, nineteen were successful, while of fifty-five cases of supra-pubic, forty-nine were attended with success. From the same table it appears that the recto-vesical puncture is less fatal, although more likely to result in fistulæ, infiltration and abscess.

The chapter on urinary deposits is very meager and unsatisfactory.

The subjects of calculus and structure are treated at length. In the appendix is a chapter on the prevalence of calculous disorders in the United States and Canada. The statistics of this affections are, valuable as it is only from a knowledge of the soil, climate, habits, food, and drinks. connected with this di-order that we can learn any thing of its etiology.

The work does honor to the author for his industry and energy.

For sale by Keen and Lee. J.

Report of the Executive Committee of the American Temperance Union, 1855.

A pamphlet of forty pages containing much valuable information respecting the present condition of the liquor struggle in New York and in other states. It gives in full the N. Y. liquor law, and also sundry legal opinions upon it.

The Indiana Journal of Medicine and Surgery. J. Jockson, M. D., T. W. Forshee, M. D, Editors: July, 1855.--Terms two dollars per annum in advance; Madison, Ind.

It seems that an effort is again being made to nurse into life an Indiana Journal. Success to it, may it live longer than its predecessor. The number before us is creditable in matter, although rather inferior in style.

